# The Ether Lipid Precursor Hexadecylglycerol Causes Major Changes in the Lipidome of HEp-2 Cells

**DOI:** 10.1371/journal.pone.0075904

**Published:** 2013-09-30

**Authors:** Jonas Bergan, Tore Skotland, Tuulia Sylvänne, Helena Simolin, Kim Ekroos, Kirsten Sandvig

**Affiliations:** 1 Centre for Cancer Biomedicine, Faculty of Medicine, University of Oslo, Oslo, Norway; 2 Department of Biochemistry, Institute for Cancer Research, The Norwegian Radium Hospital, Oslo University Hospital, Oslo, Norway; 3 Department of Biosciences, University of Oslo, Oslo, Norway; 4 Zora Biosciences, Espoo, Finland; University of Geneva, Switzerland

## Abstract

The ether-lipid precursor *sn-1*-O-hexadecylglycerol (HG) can be used to compensate for early metabolic defects in ether-lipid biosynthesis. To investigate a possible metabolic link between ether-linked phospholipids and the rest of the cellular lipidome, we incubated HEp-2 cells with HG. Mass spectrometry analysis revealed major changes in the lipidome of HG-treated cells compared to that of untreated cells or cells treated with palmitin, a control substance for HG containing an acyl group instead of the ether group. We present quantitative data for a total of 154 species from 17 lipid classes. These species are those constituting more than 2% of their lipid class for most lipid classes, but more than 1% for the ether lipids and glycosphingolipids. In addition to the expected ability of HG to increase the levels of ether-linked glycerophospholipids with 16 carbon atoms in the *sn-1* position, this precursor also decreased the amounts of glycosphingolipids and increased the amounts of ceramide, phosphatidylinositol and lysophosphatidylinositol. However, incubation with palmitin, the fatty acyl analogue of HG, also increased the amounts of ceramide and phosphatidylinositols. Thus, changes in these lipid classes were not ether lipid-dependent. No major effects were observed for the other lipid classes, and cellular functions such as growth and endocytosis were unaffected. The data presented clearly demonstrate the importance of performing detailed quantitative lipidomic studies to reveal how the metabolism of ether-linked glycerophospholipids is coupled to that of glycosphingolipids and ester-linked glycerophospholipids, especially phosphatidylinositols.

## Introduction

Membrane lipid composition is critical for cell signaling, intracellular transport and cell proliferation. Lipid rafts are enriched in cholesterol and glycosphingolipids, and seem to act as signaling platforms [1]. Moreover, phosphatidylinositolphosphates (PIPs; abbreviations of lipid classes given in Methods under the heading Annotation of lipid species) are involved in recruiting a variety of cytosolic proteins involved in endocytosis and intracellular transport [2]. Furthermore, hydrolysis of lipids by enzymes such as PLA2 is important for cellular function [3]. Knowledge about the role of single lipid species and the complex interplay between these lipids and proteins is crucial for our understanding of normal cell growth, as well as changes occurring in e.g. cancer cells. Not surprisingly, alterations in lipid composition are associated with cancer, and there is evidence that the lipids in food, for instance the content of unsaturated fat, is important for the incidence of certain cancer types [4]. Attempts are being made to treat cancer with agents that modify lipid composition. For instance, inhibitors of fatty acid synthase, which can be overexpressed in cancer, are being investigated for their ability to affect cancer growth [5]. Also, inhibiting lipid anchoring of kinases such as Ras might change cell growth [6].

**Figure 1:**
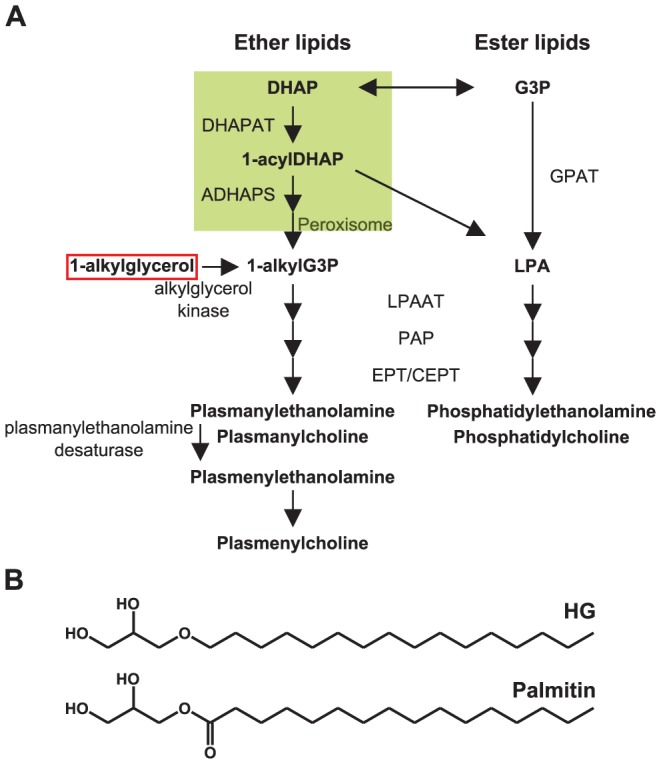
Biosynthesis of ether and ester glycerophospholipids and the chemical structures of the precursors used. (**A**) Schematic overview of the biosynthesis of ether and ester glycerophospholipids. Note that 1-alkylglycerols such as HG (red box) may enter the pathway through phosphorylation to 1-alkylglycerol 3-phosphate by an alkylglycerol kinase. For abbreviations of the compounds and enzymes, see below. (**B**) The chemical structure of the compounds used in this study, HG and palmitin. DHAP; dihydroxyacetone phosphate, G3P; glycerol 3-phoshpate, DHAPAT; dihydroxyacetone phosphate acyltransferase, GPAT; glycerol phosphate acyltransferase, ADHAPS; alkyldihydroxyacetone phosphate synthase, LPA; lysophosphatidic acid, LPAAT; lysophosphatidic acid acyltransferase, PAP; phosphatidic acid phosphatase, EPT; ethanolamine phosphotransferase, CEPT; choline/ethanolamine phosphotransferase.

Cell membranes are most often described as built up of three main lipid classes, i.e. glycerophospholipids (GPs), sphingolipids and cholesterol. By such a classification the GPs include lipids synthesized by different pathways as illustrated in Fig. 1A. The most common GPs are based on the glycerol backbone in which the hydrophobic chains are fatty acyl groups. However, also ether-containing GPs are common. They often constitute 10–20% of the total GPs in cellular membranes; and even more in certain organs, e.g. approx. 1/3 of total GPs in heart and muscle, and 1/5 of total GPs in human brain. Although ether-linked lipids have a backbone and head groups similar to GPs with fatty acyl groups only, *de novo* synthesis of ether-linked lipids starts by addition of an acyl group to dihydroxyacetonephosphate. The ether-containing lipids in mammalian cells contain an ether-linked alkyl or alkenyl group in the *sn-1* position. The species having an alkenyl group are often referred to as plasmalogens. These ether-linked lipids are so often neglected in text books and scientific articles, that they even have been called the “forgotten” lipids. For general reviews of ether-linked GPs, see [7]–[11].

Both ester-linked and ether-linked GPs consist of a mixture of different species, i.e. molecules with a different composition of fatty acids (FAs), alkyl or alkenyl groups. Remarkably, most ether-linked PE species (which most often is the dominating ether-linked GP) have an alkenyl group, whereas most of the ether-linked PC species have an alkyl group [10]–[12]. Studies performed with spin-labeled lipids show that most (70–80%) of the ether-linked PE species in the plasma membrane are localized in the inner leaflet, whereas most (70–80%) ether-linked PC species are found in the outer leaflet. Thus, the ether-linked GPs seem to have a distribution similar to the corresponding ester-linked GPs [13]. Although most ether-linked GPs are either of the PE or PC classes, also ether-linked species of other lipid classes such as PI, PS and PA as well as phosphatidylthreonines have been detected in a macrophage cell line [14].

The biological role of the ether-linked GPs remains enigmatic, although several possible functions have been discussed. The alkenyl-linked PE species are the largest endogenous providers of polyunsaturated FAs for prostanoid production and cell signaling; and a PLA2 selective for ether-linked GPs has been identified [9], [15]. Moreover, the vinyl-ether bond is sensitive to oxidation by free radicals, and there is some evidence that plasmalogens protect cells from damage by such radicals [9], [10], [16]. Furthermore, there is compelling evidence, although indirect, that alkenyl PE is critical for human health. This evidence is partly based on the identification of multiple peroxisomal disorders in which plasmalogen biosynthesis and content are severely compromised [9], [11]. Also, ether-linked GPs are major lipid constituents of several membranes undergoing a rapid membrane fusion [17]. In addition, alkenyl PE is essential for cholesterol transport from the cell surface and endocytic compartments to the ER [18]. Importantly, ether-linked lipids are required for generation of alkyl-containing glycosylphospatidylinositol (GPI)-anchored proteins by remodeling [19]. Altogether, these issues point to ether lipids as being essential for cellular functions, including intracellular transport.

The importance of ether-linked lipids has been studied in mice lacking DHAPAT, i.e. the first enzyme in the synthesis of ether-linked GPs (Fig. 1A). The ether lipid-deficient mice have several abnormalities in the CNS, and they also exhibit early postnatal bilateral cataractogenesis. Furthermore, the males become infertile and the females subfertile [8], [20], [21]. Such knock-out mice benefit from receiving a diet containing an alkylglycerol, i.e. a precursor for the ether-linked GP synthesis [17]. Alkylglycerols in the diet are also incorporated into plasmalogens in human erythrocytes and most rat tissues [22]. Also *in vitro* studies show that the cellular levels of ether-linked lipids can be restored by adding alkylglycerols [23], [24]. Moreover, alkylglycerols have been used as anti-cancer agents in animal and clinical trials [25], [26].

Recently, ether-linked GP species have been reported to be reliable markers to evaluate malignancy and metastatic capacity of human cancers. An increase in the ratio of monounsaturated to saturated species of alkenyl PC was reported for breast, lung and prostate neoplastic samples [27]. Thus, more knowledge about the ether-linked GPs is important not only for a better understanding of lipid metabolism and cellular function, but also for treatment of several diseases.

To affect cellular behavior by interfering with lipid composition, one needs detailed knowledge not only about the lipid classes in membranes, but also about the single lipid species and how their synthesis is regulated. In the present study we have used mass spectrometry (MS) to perform detailed quantitative lipidomic analyses of HEp-2 cells treated with *sn-1*-O-hexadecylglycerol (HG), i.e. a precursor for the ether-linked GPs, and the corresponding fatty acyl substance palmitin (Fig. 1B). Also untreated cells were analyzed for comparison. More than 300 species from 17 different lipid classes were quantified. As expected, HG increased the level of cellular ether-linked lipids with 16 carbon atoms in the *sn-1* position. Surprisingly, HG induced an increase of LPI (50 times) and a decrease in all glycosphingolipid classes analyzed, whereas both HG and palmitin increased the level of Cer and PI.

## Materials and Methods

### Materials

*sn-1-*O-hexadecylglycerol (HG) was from Santa Cruz Biotechnology, and dl-α-palmitin was from Sigma-Aldrich. Other chemicals used were from Sigma-Aldrich unless otherwise stated.

### Cell culture

In this study we analyzed HEp-2 cells because we have performed several studies on toxin transport in these cells. HEp-2 cells (obtained from ATCC/LGC) were grown at 5% CO_2_ in Dulbecco's modified Eagle medium (Invitrogen) supplemented with 10% (v/v) fetal calf serum (PAA Laboratories), 100 U/mL penicillin and 100 U/mL streptomycin (Invitrogen). Cells were seeded in 6-well plates 1 day prior to experiments.

### Harvesting of cells for lipidomics

For all experiments cells were treated by adding 20 µM HG (dissolved in ethanol), 20 µM palmitin (dissolved in ethanol) or 0.1% (v/v) ethanol (control) 24 hours before the cells were harvested for lipid analysis. Cells were washed in warm HEPES medium; trypsin/EDTA was added and the incubation continued at 37°C with 5% CO_2_ until the cells detached. Cells were then resuspended in HEPES medium and transferred to microfuge tubes, centrifuged for 10 min at (2500× *g*), washed with PBS and centrifuged again before freezing the cell pellet at −80°C. For determining protein content cells were lysed in 0.1 M NaCl, 10 mM Na_2_HPO_4_ (pH 7.4), 1 mM EDTA, 1% Triton-X-100, supplemented with a mixture of Complete protease inhibitors (Roche Diagnostics). The protein content was quantified using Pierce BCA protein assay kit as described by the manufacturer and using bovine serum albumin as standard protein.

### Annotations of lipid species

The different lipid species of phosphatidylcholine (PC), phosphatidylethanolamine (PE), phosphatidylserine (PS), phosphatidylinositol (PI), phosphatidic acid (PA), phosphatidylglycerol (PG) and diacylglycerol (DAG) are listed with the two fatty acyl groups separated with a slash (/) when the fatty acid positions are known (e.g. PC 16:0/16:0) and hyphen (−) when minor fractions of positional isomers may appear (e.g. PC 16:0–18:1 may include a minor fraction of PC 18:1/16:0), in accordance with IUPAC recommendations. LysoPI and lysoPC are abbreviated as LPI and LPC, respectively. The ether-linked GPs are shown as PC O (alkyl), PC P (alkenyl), PE O (alkyl) and PE P (alkenyl). The abbreviation GP is used to indicate all glycerophospholipids. The fatty acyl groups of ether-linked lipids and the N-amidated fatty acyl groups for SM, ceramide (Cer) and glycosphingolipids are shown after the slash. Abbreviations for glycosphingolipids: glucosylceramide (GlcCer), lactosylceramide (LacCer) and globotriaosylceramide (Gb3). Cholesteryl esters are abbreviated CE.

### Lipid extraction

Lipids were extracted from approximately 1.5 million cells containing 215–230 µg of protein using a modified Folch lipid extraction procedure [28]. Known amounts of the deuterium-labeled or heptadecanoyl-based synthetic internal standards; LPC 17:0, PC 17:0/17:0, PE 17:0/17:0, PS 17:0/17:0, PG 17:0/17:0, PA 17:0/17:0, Cer d18:1/17:0, SM d18:1/12:0 (Avanti Polar Lipids Inc.), DAG 17:0/17:0 (C/D/N Isotopes Inc.), D6-CE 18:0 (C/D/N Isotopes Inc.), D3-GlcCer d18:1/16:0, D3-LacCer d18:1/16:0, and Gb3 d18:1/17:0 (Matreya LLC) were added and used for quantification of the endogenous lipid species as previously described [29], [30]. The LPC standard was used to estimate the amount of all lysoPLs, whereas PI species were estimated using the PG standard. The PE and PC standards were used to estimate the amount of ether lipids with PE or PC headgroups, respectively. Following lipid extraction, samples were reconstituted in chloroform:methanol (1∶2, v/v) and stored at −20°C prior to MS analysis.

### MS analyses

The species of GPs, SM, DAG and CE were analyzed by shotgun analysis on a hybrid triple quadrupole/linear ion trap mass spectrometer (QTRAP 5500, AB SCIEX) equipped with a robotic nanoflow ion source (NanoMate HD, Advion Biosciences) [31]. Both positive and negative ion modes using multiplexed precursor ion scanning and neutral loss (NL)-based methods were used [28], [32]. The identification of ether-linked species was confirmed by sequential MS/MS analyses in negative ion mode. Collision energy of 40 eV was used. The CEs were analyzed in positive ion mode [33]. Sphingolipids were analyzed by reverse phase ultra-high pressure liquid chromatography (UHPLC) as previously described [34] using an Acquity BEH C18, 2.1×50 mm column with a particle size of 1.7 µm (Waters, Milford, Massachusetts, USA) coupled to a hybrid triple quadrupole/linear ion trap mass spectrometer (QTRAP 5500, AB SCIEX). A 25 min gradient using 10 mM ammonium acetate in water with 0.1% formic acid (mobile phase A) and 10 mM ammonium acetate in acetonitrile:2-propanol (4∶3, v/v) containing 0.1% formic acid (mobile phase B) was used. Multiple reaction monitoring (MRM) was used for quantification of sphingolipids. Identification of the ether-containing species was performed according to Hsu and Turk [35].The MS lipidomic analyses were performed in a laboratory used to work according to GLP (Good Laboratory Practice), and published validation data show less than 15% variation for most lipid species even when analyzed on three different days [30].

### Data processing

The MS data files were processed as previously described [29] using Lipid Profiler™ and MultiQuant™ software for producing a list of lipid names and peak areas. A stringent cutoff was applied for separating background noise from actual lipid peaks. Masses and counts of detected peaks were converted into a list of corresponding lipid names. Lipids were normalized to their respective internal standard [29] and the concentrations of molecular lipids are presented as pmol/µg protein. Quality control samples were utilized to monitor the overall quality of the lipid extraction and mass spectrometry analyses [30]; these samples were mainly used to remove technical outliers and lipid species that were detected below the lower limit of quantification. The data are shown as mean values of results for two independent experiments with bars showing the standard deviation. It should be noted that the ether lipids, PI and lysoPLs other than LPC are estimated based on using standards of other classes, whereas all other lipids are quantified using standards of their own class.

## Results

**Figure 2:**
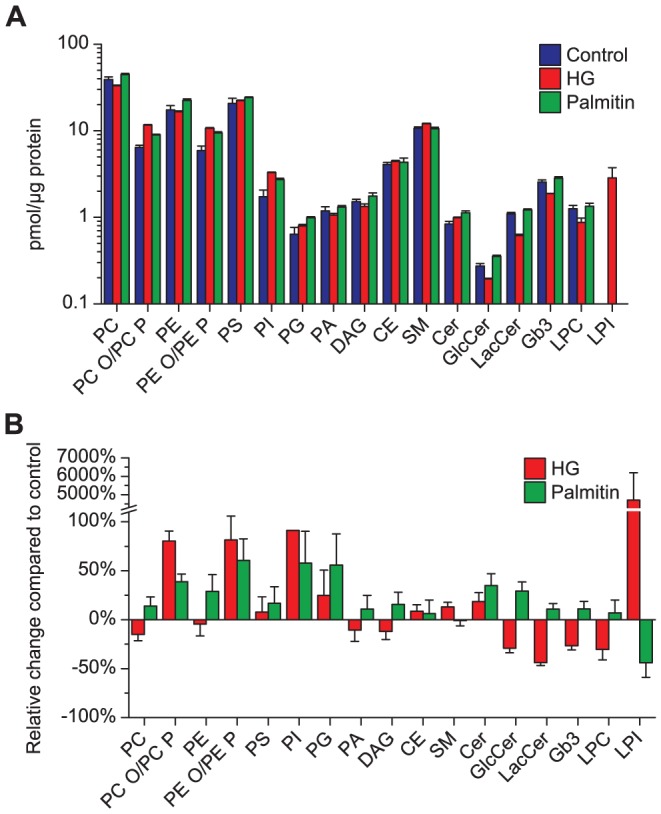
Overall changes in the lipidome of HEp-2 cells after treatment with HG or palmitin. HEp-2 cells were treated for 24 hours with HG (20 µM), palmitin (20 µM) or ethanol (0.1%, as control) before analyzing the lipidome by MS. (**A**) The total amount of the different lipid classes are shown as absolute values (note the logarithmic scale) and (**B**) the difference between treated cells and control cells are expressed as relative values.

**Figure 3:**
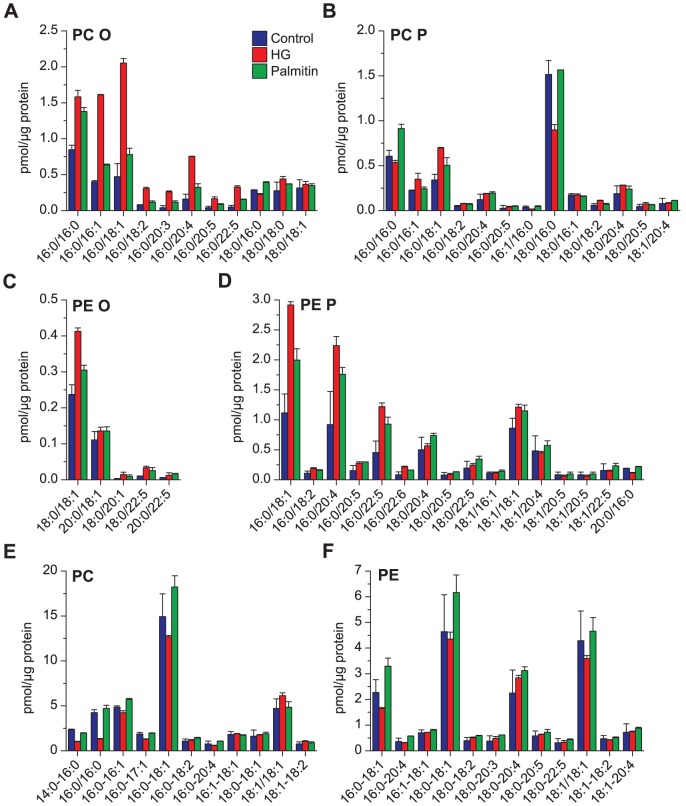
Quantitative analysis of glycerophospholipids after HG or palmitin treatment. The major species of (**A**) PC O, (**B**) PC P, (**C**) PE O, (**D**) PE P, (**E**) PC and (**F**) PE, in HEp-2 cells treated with HG (20 µM), palmitin (20 µM) or ethanol (0.1%, as control) for 24 hours. The species shown here are species comprising more than 1% of the total mass of the ether lipids and more than 2% of PC and PE for at least one of the samples.

**Figure 4:**
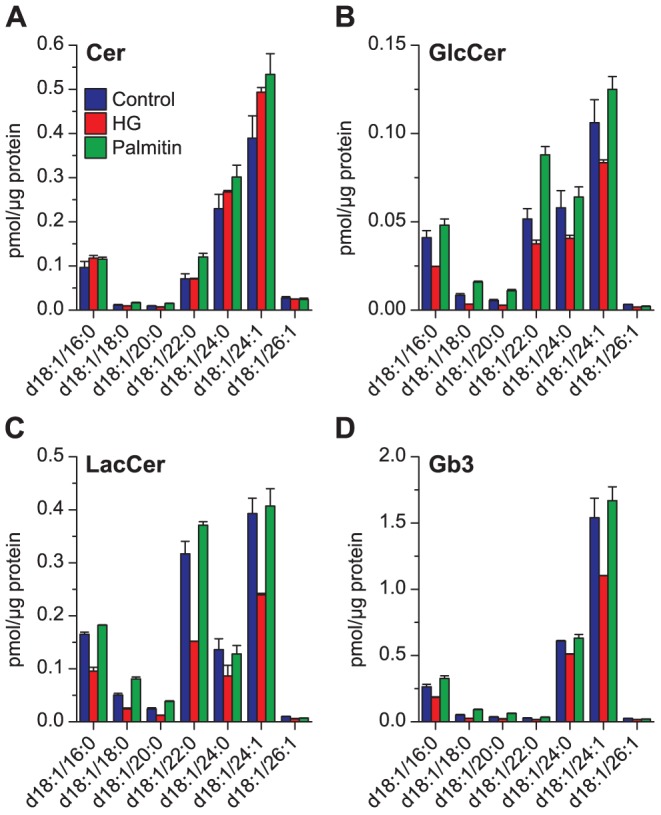
Quantitative analysis of ceramide and glycosphingolipids after HG or palmitin treatment. The major species of (**A**) Cer, (**B**) GlcCer, (**C**) LacCer, and (**D**) Gb3 in HEp-2 cells treated with HG (20 µM), palmitin (20 µM) or ethanol (0.1%, as control) for 24 hours. The species shown here are species comprising more than 1% of the total mass of any of the classes.

**Figure 5:**
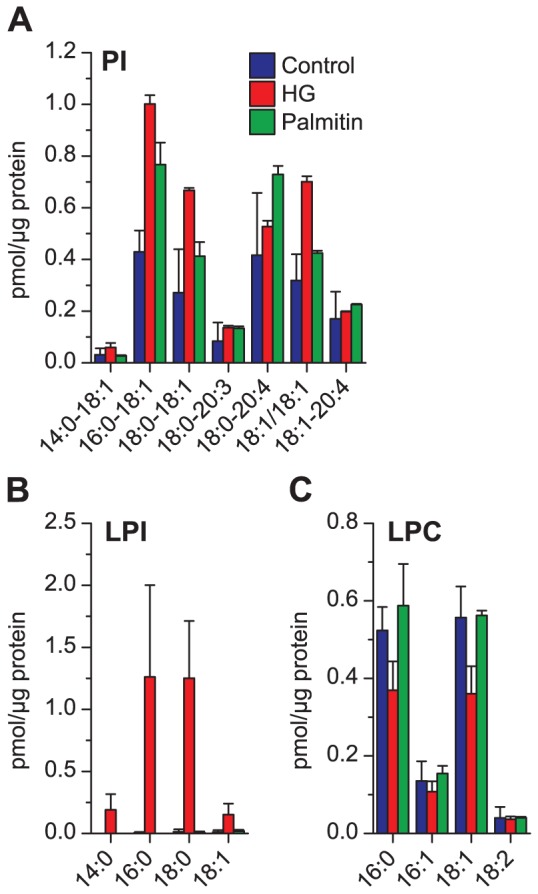
Quantitative analysis of phosphoinositide lipids and LPC after HG or palmitin treatment. The major species of (**A**) PI, (**B**) LPI, and (**C**) LPC in HEp-2 cells treated with HG (20 µM), palmitin (20 µM) or ethanol (0.1%, as control) for 24 hours. The species shown here are species comprising more than 2% of the total mass of the lipid class for at least one of the samples.

**Figure 6:**
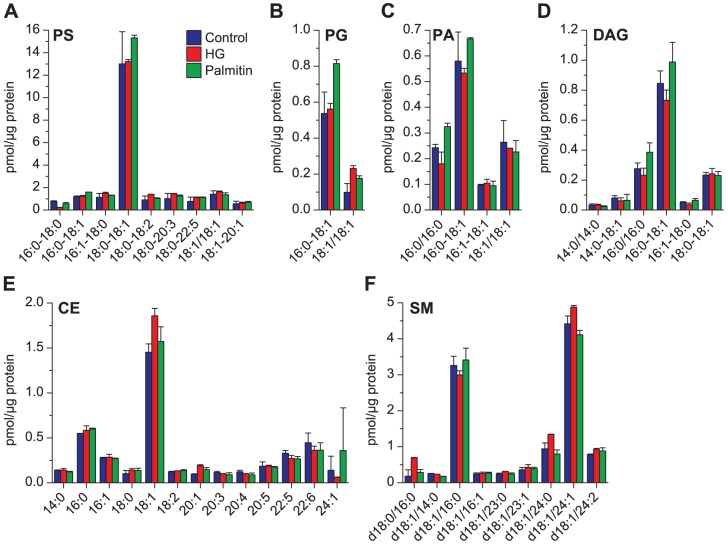
Quantitative lipid analysis of PS, PG, PA, DAG, CE and SM. The major species of (**A**) PS, (**B**) PG, (**C**) PA, (**D**) DAG, (**E**) CE, and (**F**) SM in HEp-2 cells treated with HG (20 µM), palmitin (20 µM) or ethanol (0.1%, as control) for 24 hours. The SM species labeled d18:1/23:0 and d18:1/23:1 may alternatively be d18:1//22:0(OH) and d18:1/22:1(OH). The species shown here are species comprising more than 2% of the total mass of the lipid class for at least one of the samples.

More than 300 species from 17 lipid classes were quantified following extraction of lipids from HEp-2 control cells as well as from cells treated with HG and palmitin. The analyses were performed on two independently treated samples using an analytical method that has been shown to give less than 15% variation for most lipid species [30]. Similar data were obtained in an initial experiment with control cells and cells treated with HG (two independently treated samples; palmitin not included; data not shown). A full list of all quantified species can be found in the supporting information (Dataset S1). Data for 154 species are presented in Figs. 2–6. Each of these species constitutes more than 1% of the lipids in their class for ether lipids and glycosphingolipids and more than 2% of the other classes. This was done to make the data in Figs. 2–6 better readable. We confirmed the identities of 30 species of ether-linked PCs and 26 species of ether-linked PEs by using subsequential MS/MS analyses (Dataset S1). The sum of the species quantified (pmol lipid/µg protein) are shown in Fig. 2A. The relative changes of the lipid composition of the HG or palmitin-treated cells compared to the untreated cells are shown in Fig. 2B. The changes observed for the different classes and species of each class is detailed below.

The quantification of the major phosphatidylcholine (PC) and phosphatidylethanolamine (PE) molecular species are shown in Fig. 3. The data in Fig. 3A show that there was a large increase in several PC O species with 16:0 in the *sn-1* position (PC O 16:0/16:1 and PC O 16:0/18:1 increased 300–400%) following treatment with HG. Interestingly, some of these species were also somewhat increased (approximately 50% for the two species mentioned above) following treatment with palmitin. Surprisingly, there was no strong effect on PC P species upon HG treatment (Fig. 3B), and PC P 16:0/16:0 actually decreased in contrast to PC O 16:0/16:0. One of the main species with 18:0 in the *sn-1* position (PC P 18:0/16:0) was in HG-treated cells reduced to approximately 60% of that present in untreated cells, whereas the level of this species was similar in palmitin-treated cells and untreated cells. For PC species (Fig. 3E) there was a reduction in most species with 16:0 in the *sn-1* position following treatment with HG (PC 16:0/16:0 was down to 30% of the control cells, whereas most other of these species were reduced only 15–20%). The other PC species were unchanged. Cells treated with palmitin had no dramatic effect on the molecular PC profile.

The data for ether-linked PE species (Fig. 3C, 3D) reveal a major increase (200–300%) in cells treated with HG for some PE P species with 16:0 in the *sn-1* position; also cells treated with palmitin showed increase (up to 80%) in some PE P species, i.e. these species showed a similar trend as mentioned for the PC O and PC P species. However, we did not observe any decrease of PE P species with 18:0 in the *sn-1* position as observed for PC P. PE species with 16:0 in the *sn-1* position decreased only slightly (Fig. 3F), in contrast to what we observed for PC species with 16:0 in the *sn-1* position (Fig. 3E).

An unexpected effect was observed in the levels of ceramides (Cer) and glycosphingolipids following treatment with HG and palmitin (Fig. 4). Treatment with HG resulted in increased levels of the very long chain ceramide species Cer d18:1/24:0 and Cer d18:1/24:1, and the increase was even slightly larger (up to 35–40%) in the cells treated with palmitin (Fig. 4A). In both treatments, the short chain Cer d18:1/16:0 was less affected. This profile picture changed dramatically when looking at glycosylated synthesis products of ceramides, i.e. glucosylceramides (GlcCer), lactosylceramides (LacCer) and globotriaosylceramide (Gb3). The HG treatment resulted in a major reduction in all monitored GlcCer, LacCer and Gb3 species compared to the untreated cells (Fig. 4B–D). In contrast, the cells treated with palmitin showed somewhat higher values than the untreated cells for GlcCer, but were very similar to the untreated cells for LacCer and Gb3. Thus, these data indicate that the only effect of palmitin treatment on the levels of glycosphingolipids seems to be mediated by the increased amounts of Cer, being the substrate for GlcCer synthase.

HG treatment also had a major effect on the level of several PI species, i.e. an increase of approximately 250% for major species such as PI 16:0–18:1, PI 18:0–18:1and PI 18:1/18:1 (Fig. 5A). However, treatment with palmitin also resulted in increased levels of certain PI species (40–80% for the species mentioned above). Perhaps the most remarkable effect of the HG treatment was the dramatic effect on the LPI species (Fig. 5B), which increased to more than 50 times the level of that in control cells. This effect was not a general effect on lysophospholipids as there was a small decrease of LPC (Fig. 5C), the major lysophospholipid in untreated cells. Palmitin did not have any significant effect on the level of LPC. The percent decrease of LPI shown in Fig. 2B should be considered in light of the very low amount of LPI present in control cells (Fig. 2A). The data for the major species of PS, PG, PA, DAG, CE and SM did not reveal any major changes for any of these lipid classes or species following treatment with either HG or palmitin (Fig. 6).

## Discussion

A major finding in the present study is that the alkylglycerol HG, a precursor for ether-linked GPs, gives a much larger effect on the cellular lipidome than expected. In this article, we report quantitative lipidomic analyses for 154 species of 17 lipid classes from HEp-2 control cells and from HEp-2 cells treated with HG or palmitin (a control substance for HG containing an acyl group instead of the ether group). In spite of major effects on some lipid classes following this treatment, we did not observe any changes in the ability of these cells to undergo cell division, their endocytosis of transferrin, or toxicity of the plant toxin ricin (data not shown). As ricin is endocytosed by a variety of endocytic mechanisms and transported retrogradely through the Golgi apparatus to the ER before being translocated to cytosol where it inhibits the protein synthesis [36], these data demonstrate that many cellular functions of HG-treated cells remain unchanged.

Treatment of HEp-2 cells with HG gave as expected increased levels of some ether-linked PC and PE species with 16:0 in the *sn-1* position. The treatment also resulted in a decrease of the major ether-linked PC species with 18:0 in the *sn-1* position. These changes are most likely due to the changed amount of available substrates (precursors) for lipid synthesis in cells incubated with HG.

Remarkably, treatment of HEp-2 cells with HG reduced the amounts of glycosphingolipids including GlcCer, although these cells have higher levels of Cer, i.e. the substrate for formation of GlcCer. The HG-treated cells also contained less of all species of LacCer and Gb3 than the untreated cells. The mechanism behind these changes is not understood. It should be noted that only minor changes were observed for the SM species (Fig. 6F).

Treatment of the HEp-2 cells with HG surprisingly gave increased amounts of PI and LPI, whereas no significant effects were observed for PS, PG, PA, DAG, CE and SM. More than 50 times increase was observed for LPI species following treatment with HG. The formation of the LPI species is most likely caused by the action of PLA2 on PI, although it should be mentioned that the applied MS analysis is not able to discriminate between the FAs of lysophospholipids being in the *sn-1* or *sn-2* position. Lysophospholipids have during recent years been shown to have a number of biological functions [37], and LPI has recently been suggested to be a specific ligand for the orphan G protein-coupled receptor GPR55 [38]. It should be noted that even though we did not measure phosphatidylinositolphosphates (PIPs), the data showing increased levels of PI and LPI suggest that HG treatment might result in important changes also for the PIPs, which are known to play important roles in a variety of cellular functions [2]. However, future studies are needed to understand the mechanisms behind the effects of HG on the metabolism of PI, LPI and possibly also PIPs. It is also important to keep in mind that GPI anchor remodeling depends on ether lipid biosynthesis [19].

The present data show that GPs with choline headgroups (including ether species), PG, PA and DAG species mainly have shorter FAs (C16–18) and very little of longer (C20–22) polyunsaturated FAs of those most commonly found in GPs, whereas PI, PE (including ether species) and PS have considerably more of longer polyunsaturated FAs. Although similar data have been reported in several studies [10], [39], [40], there is so far no mechanistic explanation for these differences. However, a functional consequence for the cell might be that the action of PLA2 on PE or PI (these classes are in the plasma membrane mainly localized in the inner leaflet) results in intracellular release of e.g. arachidonic acid, thus creating signaling substances.

We have earlier reported lipidomic data which suggest a connection between the levels of sphingolipids and ether-linked lipids [41]. Whereas treatment of HEp-2 cells with an inhibitor of GlcCer synthase (PDMP) gave decreased amounts of glycosphingolipids and no observed effects on other lipid classes, major changes in the lipidome were seen following treatment of the cells with Fumonisin B_1_ (FB1), an inhibitor of dihydroceramide synthase. Thus, FB1 gave increased levels of short-chain species (34 or 36 carbon atoms) of PE and ether-linked PE, whereas it did not change the level of long-chain species (40 carbon atoms or more). FB1 also decreased the amounts of many ether-linked PC species (no effect of chain length), but gave no or only minor effects on PC species. It should be mentioned, that it is not known if the effects observed following treatment with FB1 are caused by metabolic alterations after inhibition of dihydroceramide synthase or if FB1 also has an effect on other enzymes. Furthermore, the changes observed in the PE species following treatment with FB1 may be related to degradation of sphingosine to ethanolamine-phosphate and hexadecenal via sphingosine phosphate lyase.

Ford and Gross performed several studies 30 years ago, of synthesis of ether-linked lipids, including perfusion experiments where several radioactively labeled precursors were shown to be rapidly incorporated into plasmalogens in rabbit hearts, and they concluded that head group remodeling of plasmalogens was much faster than *de novo* synthesis of these lipids [42]. Recently Wood *et al.* [43] reported that a 3-substituted, 1-alkyl, 2-acyl glycerol ether could be transformed into plasmalogens *in vitro* in lymphocytes and *in vivo* in mouse tissues. However, to our knowledge, it has not previously been shown that addition of a plasmalogen precursor results in changes of the metabolism of glycosphingolipids and PI/LPI.

Our data demonstrate the importance of performing quantitative lipidomics when studying the effect of addition of lipid precursors. From the data presented, it is clear that the levels of different ester-linked and ether-linked GP species as well as glycosphingolipids are controlled in a complicated way that we do not yet understand. The large improvement in MS analyses of lipids during the last years will hopefully within the next few years increase our knowledge about how synthesis and degradation of the different lipid classes and species are controlled. We strongly believe that to increase our understanding of cellular lipid metabolism we should not continue to include ester-linked and ether-linked GPs in one class, only depending upon the head-group of the lipids. The fact that they have two very different pathways for biosynthesis, and the data presented in the present work, stress the importance of thinking about ether-linked and ester-linked GPs as completely different classes, in spite of their common head groups.

## Supporting Information

Dataset S1 **Quantified lipid species.**
(XLSX)

## References

[1] Simons K, Toomre D (2000) Lipid rafts and signal transduction. Nat Rev Mol Cell Biol 1: 31–39 doi:10.1038/35036052 11413487 10.1038/35036052

[2] Mayinger P (2012) Phosphoinositides and vesicular membrane traffic. Biochim Biophys Acta 1821: 1104–1113 doi:10.1016/j.bbalip.2012.01.002 22281700 10.1016/j.bbalip.2012.01.002 PMC3340507

[3] Burke JE, Dennis EA (2009) Phospholipase A2 structure/function, mechanism, and signaling. J Lipid Res 50 (Suppl) S237–242 doi:10.1194/jlr.R800033-JLR200 19011112 10.1194/jlr.R800033-JLR200 PMC2674709

[4] Murphy RA, Mourtzakis M, Mazurak VC (2012) n-3 polyunsaturated fatty acids: the potential role for supplementation in cancer. Curr Opin Clin Nutr Metab Care 15: 246–251 doi:10.1097/MCO.0b013e328351c32f 22366922 10.1097/MCO.0b013e328351c32f

[5] Menendez JA, Lupu R (2007) Fatty acid synthase and the lipogenic phenotype in cancer pathogenesis. Nat Rev Cancer 7: 763–777 doi:10.1038/nrc2222 17882277 10.1038/nrc2222

[6] Baines AT, Xu D, Der CJ (2011) Inhibition of Ras for cancer treatment: the search continues. Future Med Chem 3: 1787–1808 doi:10.4155/fmc.11.121 22004085 10.4155/fmc.11.121 PMC3347641

[7] Brites P, Waterham HR, Wanders RJA (2004) Functions and biosynthesis of plasmalogens in health and disease. Biochim Biophys Acta 1636: 219–231 doi:10.1016/j.bbalip.2003.12.010 15164770 10.1016/j.bbalip.2003.12.010

[8] Gorgas K, Teigler A, Komljenovic D, Just WW (2006) The ether lipid-deficient mouse: tracking down plasmalogen functions. Biochim Biophys Acta 1763: 1511–1526 doi:10.1016/j.bbamcr.2006.08.038 17027098 10.1016/j.bbamcr.2006.08.038

[9] Bakovic M, Fullerton MD, Michel V (2007) Metabolic and molecular aspects of ethanolamine phospholipid biosynthesis: the role of CTP:phosphoethanolamine cytidylyltransferase (Pcyt2). Biochem Cell Biol 85: 283–300 doi:10.1139/o07-006 17612623 10.1139/o07-006

[10] Wallner S, Schmitz G (2011) Plasmalogens the neglected regulatory and scavenging lipid species. Chem Phys Lipids 164: 573–589 doi:10.1016/j.chemphyslip.2011.06.008 21723266 10.1016/j.chemphyslip.2011.06.008

[11] Braverman NE, Moser AB (2012) Functions of plasmalogen lipids in health and disease. Biochim Biophys Acta 1822: 1442–1452 doi:10.1016/j.bbadis.2012.05.008 22627108 10.1016/j.bbadis.2012.05.008

[12] Lee TC (1998) Biosynthesis and possible biological functions of plasmalogens. Biochim Biophys Acta 1394: 129–145 doi:10.1016/S0005-2760(98)00107-6 9795186 10.1016/s0005-2760(98)00107-6

[13] Fellmann P, Hervé P, Devaux PF (1993) Transmembrane distribution and translocation of spin-labeled plasmalogens in human red blood cells. Chem Phys Lipids 66: 225–230 doi:10.1016/0009-3084(93)90010-Z 8111935 10.1016/0009-3084(93)90010-z

[14] Ivanova PT, Milne SB, Brown HA (2010) Identification of atypical ether-linked glycerophospholipid species in macrophages by mass spectrometry. J Lipid Res 51: 1581–1590 doi:10.1194/jlr.D003715 19965583 10.1194/jlr.D003715 PMC3035522

[15] Ong WY, Farooqui T, Farooqui AA (2010) Involvement of cytosolic phospholipase A(2), calcium independent phospholipase A(2) and plasmalogen selective phospholipase A(2) in neurodegenerative and neuropsychiatric conditions. Curr Med Chem 17: 2746–2763 doi:10.2174/092986710791859289 20586719 10.2174/092986710791859289

[16] Lessig J, Fuchs B (2009) Plasmalogens in biological systems: their role in oxidative processes in biological membranes, their contribution to pathological processes and aging and plasmalogen analysis. Curr Med Chem 16: 2021–2041 doi:10.2174/092986709788682164 19519379 10.2174/092986709788682164

[17] Brites P, Ferreira AS, da Silva TF, Sousa VF, Malheiro AR, et al (2011) Alkyl-glycerol rescues plasmalogen levels and pathology of ether-phospholipid deficient mice. PLoS ONE 6: e28539 doi:10.1371/journal.pone.0028539 22163031 10.1371/journal.pone.0028539 PMC3232224

[18] Munn NJ, Arnio E, Liu D, Zoeller RA, Liscum L (2003) Deficiency in ethanolamine plasmalogen leads to altered cholesterol transport. J Lipid Res 44: 182–192 doi:10.1194/jlr.M200363-JLR200 12518037 10.1194/jlr.m200363-jlr200

[19] Kanzawa N, Maeda Y, Ogiso H, Murakami Y, Taguchi R, et al (2009) Peroxisome dependency of alkyl-containing GPI-anchor biosynthesis in the endoplasmic reticulum. Proc Natl Acad Sci USA 106: 17711–17716 doi:10.1073/pnas.0904762106 19815513 10.1073/pnas.0904762106 PMC2764918

[20] Rodemer C, Thai TP, Brugger B, Kaercher T, Werner H, et al (2003) Inactivation of ether lipid biosynthesis causes male infertility, defects in eye development and optic nerve hypoplasia in mice. Hum Mol Genet 12: 1881–1895 doi:10.1093/hmg/ddg191 12874108 10.1093/hmg/ddg191

[21] Da Silva TF, Sousa VF, Malheiro AR, Brites P (2012) The importance of ether-phospholipids: A view from the perspective of mouse models. Biochim Biophys Acta 1822: 1501–1508 doi:10.1016/j.bbadis.2012.05.014 22659211 10.1016/j.bbadis.2012.05.014

[22] Das AK, Holmes RD, Wilson GN, Hajra AK (1992) Dietary ether lipid incorporation into tissue plasmalogens of humans and rodents. Lipids 27: 401–405 doi:10.1007/BF02536379 1630273 10.1007/BF02536379

[23] Schrakamp G, Schalkwijk CG, Schutgens RB, Wanders RJ, Tager JM, et al (1988) Plasmalogen biosynthesis in peroxisomal disorders: fatty alcohol versus alkylglycerol precursors. J Lipid Res 29: 325–334. 3379344

[24] Styger R, Wiesmann UN, Honegger UE (2002) Plasmalogen content and beta-adrenoceptor signalling in fibroblasts from patients with Zellweger syndrome. Effects of hexadecylglycerol. Biochim Biophys Acta 1585: 39–43 doi:10.1016/S1388-1981(02)00320-7 12457713 10.1016/s1388-1981(02)00320-7

[25] Iannitti T, Palmieri B (2010) An update on the therapeutic role of alkylglycerols. Mar Drugs 8: 2267–2300 doi:10.3390/md8082267 20948908 10.3390/md8082267 PMC2953404

[26] Deniau AL, Mosset P, Le Bot D, Legrand AB (2011) Which alkylglycerols from shark liver oil have anti-tumour activities? Biochimie 93: 1–3 doi:10.1016/j.biochi.2009.12.010 20036307 10.1016/j.biochi.2009.12.010

[27] Smith RE, Lespi P, Di Luca M, Bustos C, Marra FA, et al (2008) A reliable biomarker derived from plasmalogens to evaluate malignancy and metastatic capacity of human cancers. Lipids 43: 79–89 doi:10.1007/s11745-007-3133-6 18046593 10.1007/s11745-007-3133-6

[28] Ekroos K, Chernushevich IV, Simons K, Shevchenko A (2002) Quantitative profiling of phospholipids by multiple precursor ion scanning on a hybrid quadrupole time-of-flight mass spectrometer. Anal Chem 74: 941–949 doi:10.1021/ac015655c 11924996 10.1021/ac015655c

[29] Ejsing CS, Duchoslav E, Sampaio J, Simons K, Bonner R, et al (2006) Automated identification and quantification of glycerophospholipid molecular species by multiple precursor ion scanning. Anal Chem 78: 6202–6214 doi:10.1021/ac060545x 16944903 10.1021/ac060545x

[30] Jung HR, Sylvänne T, Koistinen KM, Tarasov K, Kauhanen D, et al (2011) High throughput quantitative molecular lipidomics. Biochim Biophys Acta 1811: 925–934 doi:10.1016/j.bbalip.2011.06.025 21767661 10.1016/j.bbalip.2011.06.025

[31] Ståhlman M, Ejsing CS, Tarasov K, Perman J, Borén J, et al (2009) High-throughput shotgun lipidomics by quadrupole time-of-flight mass spectrometry. J Chromatogr B Analyt Technol Biomed Life Sci 877: 2664–2672 doi:10.1016/j.jchromb.2009.02.037 10.1016/j.jchromb.2009.02.037 19286428

[32] Ekroos K, Ejsing CS, Bahr U, Karas M, Simons K, et al (2003) Charting molecular composition of phosphatidylcholines by fatty acid scanning and ion trap MS3 fragmentation. J Lipid Res 44: 2181–2192 doi:10.1194/jlr.D300020-JLR200 12923235 10.1194/jlr.D300020-JLR200

[33] Liebisch G, Binder M, Schifferer R, Langmann T, Schulz B, et al (2006) High throughput quantification of cholesterol and cholesteryl ester by electrospray ionization tandem mass spectrometry (ESI-MS/MS). Biochim Biophys Acta 1761: 121–128 doi:10.1016/j.bbalip.2005.12.007 16458590 10.1016/j.bbalip.2005.12.007

[34] Merrill AH Jr, Sullards MC, Allegood JC, Kelly S, Wang E (2005) Sphingolipidomics: high-throughput, structure-specific, and quantitative analysis of sphingolipids by liquid chromatography tandem mass spectrometry. Methods 36: 207–224 doi:10.1016/j.ymeth.2005.01.009 15894491 10.1016/j.ymeth.2005.01.009

[35] Hsu FF, Turk J (2009) Electrospray ionization with low-energy collisionally activated dissociation tandem mass spectrometry of glycerophospholipids: mechanisms of fragmentation and structural characterization. J Chromatogr B Analyt Technol Biomed Life Sci 877: 2673–2695 doi:10.1016/j.jchromb.2009.02.033 10.1016/j.jchromb.2009.02.033 PMC2723218 19269264

[36] Sandvig K, Torgersen ML, Engedal N, Skotland T, Iversen TG (2010) Protein toxins from plants and bacteria: probes for intracellular transport and tools in medicine. FEBS Lett 584: 2626–2634 doi:10.1016/j.febslet.2010.04.008 20385131 10.1016/j.febslet.2010.04.008

[37] Grzelczyk A, Gendaszewska-Darmach E (2013) Novel bioactive glycerol-based lysophospholipids: New data - New insight into their function. Biochimie 95: 667–679 doi:10.1016/j.biochi.2012.10.009 23089136 10.1016/j.biochi.2012.10.009

[38] Piñeiro R, Falasca M (2012) Lysophosphatidylinositol signalling: new wine from an old bottle. Biochim Biophys Acta 1821: 694–705 doi:10.1016/j.bbalip.2012.01.009 22285325 10.1016/j.bbalip.2012.01.009

[39] Pike LJ, Han X, Chung KN, Gross RW (2002) Lipid rafts are enriched in arachidonic acid and plasmenylethanolamine and their composition is independent of caveolin-1 expression: a quantitative electrospray ionization/mass spectrometric analysis. Biochemistry 41: 2075–2088 doi:10.1021/bi0156557 11827555 10.1021/bi0156557

[40] Llorente A, Skotland T, Sylvänne T, Kauhanen D, Rog T, et al (2013) Molecular Lipidomics of Exosomes Released by PC-3 Prostate Cancer Cells. Biochim Biophys Acta doi:10.1016/j.bbalip.2013.04.011 10.1016/j.bbalip.2013.04.011 24046871

[41] Raa H, Grimmer S, Schwudke D, Bergan J, Wälchli S, et al (2009) Glycosphingolipid requirements for endosome-to-Golgi transport of Shiga toxin. Traffic 10: 868–882 doi:10.1111/j.1600-0854.2009.00919.x 19453975 10.1111/j.1600-0854.2009.00919.x

[42] Ford DA, Gross RW (1994) The discordant rates of sn-1 aliphatic chain and polar head group incorporation into plasmalogen molecular species demonstrate the fundamental importance of polar head group remodeling in plasmalogen metabolism in rabbit myocardium. Biochemistry 33: 1216–1222 doi:10.1021/bi00171a022 8110753 10.1021/bi00171a022

[43] Wood PL, Khan MA, Smith T, Ehrmantraut G, Jin W, et al (2011) *In vitro* and *in vivo* plasmalogen replacement evaluations in rhizomelic chrondrodysplasia punctata and Pelizaeus-Merzbacher disease using PPI-1011, an ether lipid plasmalogen precursor. Lipids Health Dis 10: 182 doi:10.1186/1476-511X-10-182 22008564 10.1186/1476-511X-10-182 PMC3238230

